# Morphometric, Nutritional, and Phytochemical Characterization of Eugenia (*Syzygium paniculatum* Gaertn): A Berry with Under-Discovered Potential

**DOI:** 10.3390/foods14152633

**Published:** 2025-07-27

**Authors:** Jeanette Carrera-Cevallos, Christian Muso, Julio C. Chacón Torres, Diego Salazar, Lander Pérez, Andrea C. Landázuri, Marco León, María López, Oscar Jara, Manuel Coronel, David Carrera, Liliana Acurio

**Affiliations:** 1 School of Chemical Sciences and Engineering, Yachay Tech University, Urcuquí 100115, Ecuador; christian.muso@yachaytech.edu.ec; 2 School of Physical Sciences and Nanotechnology, Yachay Tech University, Urcuquí 100115, Ecuador; jchacon@yachaytech.edu.ec; 3 G+ BioFood & Engineering Research Group, Department of Science and Engineering in Food and Biotechnology, Universidad Técnica de Ambato. Av. Los Chasquis & Río Payamino, Ambato 180150, Ecuador; dm.salazar@uta.edu.ec (D.S.); lv.perez@uta.edu.ec (L.P.); 4 Circular Engineering, Applied Sciences & Simulation Group (GICAS), Chemical Engineering Department, Universidad San Francisco de Quito USFQ, Diego de Robles y Vía Interoceánica, Quito 170901, Ecuador; alandazuri@usfq.edu.ec (A.C.L.); mleond@usfq.edu.ec (M.L.); 5 Thermal Spray Center (CPT), University of Barcelona, Martí i Franqués 1, 08028 Barcelona, Spain; 6 School of Agricultural and Environmental Sciences, Ibarra Campus, Pontifical Catholic University of Ecuador, Jorge Guzmán Rueda Avenue and Padre Aurelio Espinosa Polit Avenue, Ibarra 100112, Ecuador; mflopez2@pucesi.edu.ec; 7 School of Social and Human Sciences, Ibarra Campus, Pontifical Catholic University of Ecuador, Jorge Guzmán Rueda Avenue and Padre Aurelio Espinosa Polit Avenue, Ibarra 100112, Ecuador; ojara@pucesi.edu.ec; 8 School of Agricultural and Agroindustrial Sciences, Yachay Experimental Technology Research University, Urcuquí100115, Ecuador; mcoronel@yachaytech.edu.ec; 9 Ingeniería en Telecomunicaciones, Universidad de las Americas, Quito 170513, Ecuador; david.carrera.cevallos@udla.edu.ec

**Keywords:** *Syzygium paniculatum* Gaertn, *Eugenia berry*, antioxidant activity, phenolic compounds, bioactive compounds

## Abstract

Magenta Cherry or Eugenia *(Syzygium paniculatum* Gaertn) is an underutilized berry species with an interesting source of functional components. This study aimed to evaluate these berries’ morphometric, nutritional, and phytochemical characteristics at two ripening stages, CM: consumer maturity (CM) and OM: over-maturity. Morphometric analysis revealed size and weight parameters comparable to commercial berries such as blueberries. Fresh fruits were processed into pulverized material, and in this, a proximate analysis was evaluated, showing high moisture content (88.9%), dietary fiber (3.56%), and protein (0.63%), with negligible fat, indicating suitability for low-calorie diets. Phytochemical screening by HPLC identified gallic acid, chlorogenic acid, hydroxycinnamic acid, ferulic acid, quercetin, rutin, and condensed tannins. Ethanol extracts showed stronger bioactive profiles than aqueous extracts, with significant antioxidant capacity (up to 803.40 µmol _Trolox_/g via Ferric Reducing Antioxidant Power (FRAP assay). Fourier-transform infrared spectroscopy (FTIR) and Raman spectroscopic analyses established structural transformations of hydroxyl, carbonyl, and aromatic groups associated with ripening. These changes were supported by observed variations in anthocyanin and flavonoid contents, both higher at the CM stage. A notable pigment loss in OM fruits could be attributed to pH changes, oxidative degradation, enzymatic activity loss, and biotic stressors. Antioxidant assays (DPPH, ABTS, and FRAP) confirmed higher radical scavenging activity in CM-stage berries. Elemental analysis identified minerals such as potassium, calcium, magnesium, iron, and zinc, although in moderate concentrations. In summary, *Syzygium paniculatum* Gaertn fruit demonstrates considerable potential as a source of natural antioxidants and bioactive compounds. These findings advocate for greater exploration and sustainable use of this native berry species in functional food systems.

## 1. Introduction

One of the most critical challenges for the industry and researchers is finding new sources of nutritional, functional, and natural components to develop healthier products with reduced synthetic additives [1]. This pursuit has directed attention toward underexplored plant species with potential health-promoting properties [2]. In this sense, fruits, vegetables, Andean crops, plants, or parts of plant species have gained significant relevance due to their multiple benefits for human beings [3,4], particularly those containing bioactive compounds with antioxidant, anti-inflammatory, and antimicrobial properties [5]. Foods contain various ingredients rich in bioactive compounds such as lipids, peptides, and antioxidants, which are essential for human nutrition [6]. Efforts to improve the functional qualities of these natural components have demanded extensive research in the food sector, giving rise to the concept of “functional foods” [7]. The study of antioxidants in underused foods has become crucial in developing nutritional foods to prevent chronic diseases and maintain overall well-being [8]. This trend concurs with the increasing demand for “clean label” products developed with plant-derived ingredients that provide functional advantages while exerting minimal environmental impact [9].

**Figure 1:**
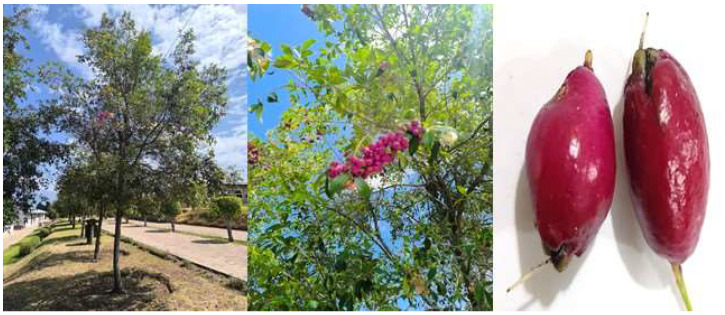
Plant and berries of *Syzygium paniculatum* Gaertn.

The species *Syzygium paniculatum* Gaertn (SPF), also named Magenta Cherry or Eugenia (in Colombia) (Figure 1), belonging to the Myrtaceae family, is known for its unique botanical characteristics [10]. According to De Amorin et al. [11], this family generally includes various species of plants with notable medicinal properties (dysentery, gastroenteritis, stomachache, and diabetes). Native to eastern Australia’s coastal regions, this tree species plays a crucial ecological role in preserving biodiversity. Its adaptability enables it to thrive in forests and cultivated areas, making it valuable for reforestation, erosion control, and urban landscaping [12]. Its resilience also supports ecological restoration by creating habitats for wildlife and stabilizing ecosystems [13]. Traditionally enjoyed in Indigenous Australian cuisines, the fruit is valued for its vibrant color, tart–sweet flavor, and versatility in various products [14]. These berries are also eaten fresh and cooked; they are currently in the form of liqueurs, wines, jams, or ice creams, to which they give a characteristic acidic flavor, showcasing their potential for modern food market commercialization [15,16]. *Syzygium paniculatum* Gaertn species is marketed in Ecuador and other countries in the region as a shrub, and its attractively colored fruits are often consumed by some birds [17]. The potential health benefits and other nutritional properties have not yet been developed in depth.

The search for natural antioxidants is vital in health sciences due to their role in combating oxidative stress linked to aging and chronic diseases [18]. In this sense, SPF has shown potential as a significant antioxidant source. Some research reports highlight the nutritional and functional value of the fruit, rich in anthocyanins, phenolics, and flavonoids with antioxidant properties that may reduce the risk of oxidative stress-related diseases or anticancer benefits, particularly for pancreatic cancer [15,19]. Also, these fruits hold notable ethnopharmacological value in Indian traditional medicine, being used to address conditions like diabetes, hyperlipidemia, hypertension, and cardiovascular issues [20]. Exploring its chemical composition and bioactivity could provide interesting insights for functional applications, advancing science and enabling the creation of health-promoting products that boost their ecological and economic value [4,21]. Therefore, SPF’s pulverized material could be added to the processing of novelty products, showcasing its versatility and market potential. These products will often present an increased nutritional value, becoming a good alternative to raw materials in the food industry. The objective of the present study was to evaluate SPF’s morphometric, nutritional, and phytochemical properties.

There are reports on the phytochemical content of SPF, but no studies were found that comprehensively characterize its morphometric, nutritional, and antioxidant capacity. This study adopts precisely that approach by connecting physical attributes with biochemical properties, thus providing a comprehensive understanding of the value of this little-known fruit. Unlike other studies, which focus on specific compounds, this work seeks to correlate structural characteristics with functional potential, thereby opening new avenues for application in the development of functional foods and nutraceutical production.

## 2. Materials and Methods

### 2.1. Raw SPF Berries Characterization

In the first stage of the research, freshly picked raw berries of *Syzygium paniculatum* Gaertn fruit (SPF) were immediately characterized to obtain basic information. The Biological Branch, Bundessortenamt und Chemische Industrie Scale (BBCH), was used for the ripening stage classification. This scale evaluates the phenology of fruit trees and has been used to describe the changes that occur in fruits and vegetables. The berries were classified into two stages of maturity: (1) consumer maturity (CM)—ripening stage 4 with 100% magenta color (Munsell color system 5 RP 4/8 descriptor intense red–purple) and epicarp with time from anthesis of 14 weeks and (2) over-maturity (OM)—ripening stage 5 with purple color (Munsell color system 5P 2/6 dark purple) and epicarp with time from anthesis of 18 weeks [10]. The SPF berries were obtained from Hacienda San Eloy, Innopolis agro-industrial park of Yachay Tech University in Urcuqui, Ecuador (0°25′12.6″ N 78°11′23.7″ W).

#### 2.1.1. Physicochemical Characterization

The dimensions, volume, shape, and weight of 200 SPF berries were evaluated. The length (L) and the width (W) of the berries were measured with a digital caliper (Neiko 01407A, Taipei, Taiwan), and the samples were weighed with a precision scale (±0.001 g) (Mettler Toledo, Greifensee, Switzerland). The berry volume was estimated as an ellipsoid volume using the formula V = 4/3πLW^2^, and the berry shape was calculated as the ratio between the length and width of the berry. Soluble solids were measured in Brix degrees in previously homogenized samples with a refractometer (Zeiss, ATAGO model NAR-3T refractometer, Tokyo, Japan) at 20 °C. The pH was determined following the Salazar, Arancibia, Ocaña, Rodríguez-Maecker, Bedón, López-Caballero and Montero [2] method. All tests were carried out in triplicate using 20 berries.

#### 2.1.2. Color

The optical properties were measured in a Minolta spectrophotometer (CM-3600d, Tokyo, Japan) with D65 standard light and observer 10. The results were expressed in CIE*L*a*b* color coordinates (spectra 400–700 nm), and chroma (C* saturation) and hue (h* tone) were also analyzed. This analysis was performed on 200 berries.

### 2.2. Production of Pulverized SPF Berries

In the second stage of the research, berries were transformed into pulverized material. The berries were washed with water until all the impurities found on the surface were eliminated. Then, the berries were cut into two parts and dried at 65 °C for eight hours in a convective oven dryer (Gander MTN, Saint Paul, MN, USA). Finally, the dried slices were ground at three 10 s intervals using an electric mill (Hamilton Beach, model: 80,393, Picton, ON, Canada) [22]. All samples were hermetically packed and stored at room temperature until further analysis.

### 2.3. Pulverized SPF Berries Characterization

#### 2.3.1. Proximate Composition

The AOAC (2023) methods [23] were used to determine moisture (920.151), total solids (920.151), fat (2003.06), ash (923.03), total dietary fiber by gravimetric–enzymatic method (985.29), total protein by Kjeldhal (991.2), and total carbohydrates by calculation. Environmental conditions during the determinations were controlled at 22.8 °C and 53% relative humidity. The reported results presented in this section are the average of three measurements.

#### 2.3.2. Scanning Electron Microscopy (SEM) Analysis

The morphometric characterization of the samples was conducted using a scanning electron microscope (SEM, JEOL JSM-IT300, JEOL Ltd., Tokyo, Japan) to analyze the surface topography and microstructural features. A thin layer of gold (Au) was sputter-coated onto non-conductive specimens using a sputter coater (Cressington 108 Auto, Ted Pella, Redding, CA, USA) to enhance conductivity and minimize charging effects. The SEM analysis was performed under high vacuum conditions at an accelerating voltage of 20 kV, with a working distance of approximately 10 mm. Secondary electron (SE) and imaging modes were employed at different magnifications to obtain high-resolution micrographs, providing insight into the surface morphology and compositional contrast, respectively.

#### 2.3.3. Phytochemical Analysis

##### High-Performance Liquid Chromatography (HPLC) Assay

A System Suitability Test (SST) was performed before the analysis to check the instrument’s performance. The calibration curves were made with gallic acid, chlorogenic acid, and quercetin standards. Each standard compound was first dissolved in methanol to prepare a mother solution at 1000 mg/L, and serial dilutions were made to obtain standards at concentrations of 10, 20, 40, 60, 80, and 100 mg/L. These standards were injected into the HPLC system (254 nm) under the same chromatographic conditions applied to the samples.

Two extracts were prepared: SPF EtOH, as the ethanolic extract, and SPF H_2_O, as the water extract. The maceration of the pulverized SPF berries was performed in ethanol (1:10 *w*/*v*) for 72 h, followed by filtration. A C18 reverse-phase column (250 mm × 4.6 mm, 5 µm) was used with a mobile phase of a gradient of water (0.1% formic acid) and acetonitrile (ACN, 0.1% formic acid) at a flow rate of 1.0 mL/min. The detection was made at a wavelength of 254 nm (UV-VIS). The ethanolic extract was injected using a volume of 5.0 µL, while the water extract was injected using 10.0 µL. The run time was 28 min for SPF EtOH and 30 min for SPF H_2_O.

##### Phytochemical Compounds Extraction

The phytochemical compounds of SPF were extracted using the Soxhlet extraction method [24]. The solvent used for the extraction was 400 mL of ethanol at 96% and 20 g of pulverized material. The final concentrated extract was collected in amber glass vials with screw caps to protect it from light and stored at 4 °C, wrapped in aluminum foil to ensure its stability for subsequent analyses (total phenolic content, antioxidant activity, flavonoid content, and anthocyanin content).

##### Total Phenolic Content (TPC) Assay

The Folin–Ciocalteu method reported by Martin et al. [25] was employed to quantify the phenolic content. A gallic acid calibration curve was prepared, and results were expressed as milligrams of gallic acid equivalents per gram of fresh weight (mg_GAE_/g_FW_).

##### Antioxidant Activity

a. - DPPH (2.2-Diphenyl-1-picrylhydrazyl) Assay

The DPPH (2,2-Diphenyl-1-picrylhydrazyl) assay, reported by Rhaman et al. [26], was conducted to measure the free radical scavenging activity of the extracts. A methanolic solution of DPPH was mixed with the extract, and the decrease in absorbance at 517 nm was monitored. Results were expressed as inhibition percentages.

b. ABTS [2,2′-Azino-bis (3-ethylbenzothiazoline-6-sulfonic acid)] Assay

The ABTS [2,2′-Azino-bis(3-ethylbenzothiazoline-6-sulfonic acid)] assay was used to evaluate hydrophilic and lipophilic antioxidant capacities, according to the method reported by Elhasannen et al. [27]. ABTS radicals were generated by reacting ABTS stock solution with potassium persulfate, and the reaction mixture was incubated in the dark for 16 h. The extracts were added, and the reduction in absorbance at 734 nm was recorded. Trolox was used as the standard, and results were expressed as inhibition percentages.

c. FRAP (Ferric Reducing Ability of Plasma) Assay

The FRAP assay was conducted by combining the extract with a ferric-TPTZ reagent, and the absorbance was measured at 593 nm after a 30-min incubation. Trolox was used as the standard, and the results were expressed as µmol of Trolox Equivalent Antioxidant Capacity (TEAC) per gram [27,28].

##### Flavonoid Content

The aluminum chloride colorimetric assay described by Shraim et al. [28] was used to determine total flavonoids, with quercetin as the standard. Results were expressed as milligrams of quercetin equivalents per gram of fresh weight (mg _QE_/g_FW_).

##### Anthocyanin Content

The High-Performance Liquid Chromatography (HPLC) at 520 nm method reported by Filip et al. [29] was used. A C18 column, a mobile phase of acetonitrile, and 0.1% formic acid were employed to identify and quantify anthocyanins.

#### 2.3.4. Spectroscopy

##### Fourier-Transform Infrared (FTIR) Spectroscopy

A Fourier-transform infrared (FTIR) spectrometer (Spectrum two, Model LI600401, CT, USA) was used to identify functional groups. Pulverized SPF berries were mixed with KBr to form pellets, and spectra were recorded in the 400–4000 cm^−1^ range. Key functional groups associated with bioactive compounds, such as hydroxyl (-OH), carbonyl (C=O), and aromatic (C=C) groups, were analyzed for shifts in intensity and wavenumber.

##### RAMAN Spectroscopy

The technique is susceptible to vibrations associated with aromatic and glycosylated compounds, which makes it ideal for analyzing phenolics and flavonoids. A Raman spectrophotometer (LabRAM HR Evolution, Horiba France SAS, Villeneuve d’Ascq, France) was used for all the measurements. Key vibrations in the Raman spectra were observed around 1600 cm^−^^1^, corresponding to C=C aromatic stretching, and 1200–1300 cm^−^^1^, associated with C-O-C glycosidic bonds.

#### 2.3.5. Elemental Analysis by Atomic Absorption Spectroscopy (AAS)

An atomic absorption spectrometer (Analytik Jena, model ContrAA 700, Jena, Germany) was employed to analyze oligo-elements in *Syzygium paniculatum* Gaertn berries.

### 2.4. Statistical Analysis

Results were analyzed by Student’s *t*-test for paired means with a significance level = 0.05. The differences are explained as mean values (m) and standard deviations (sd). The color results were analyzed by one-way ANOVA with Tukey’s multiple comparison test, with a significance level = 0.05. Both analyses used GraphPad Prism v5.03 (GraphPad Software, San Diego, CA, USA).

## 3. Results and Discussion

### 3.1. Raw SPF Berries Characterization

#### 3.1.1. Physicochemical Characterization

**Figure 2:**
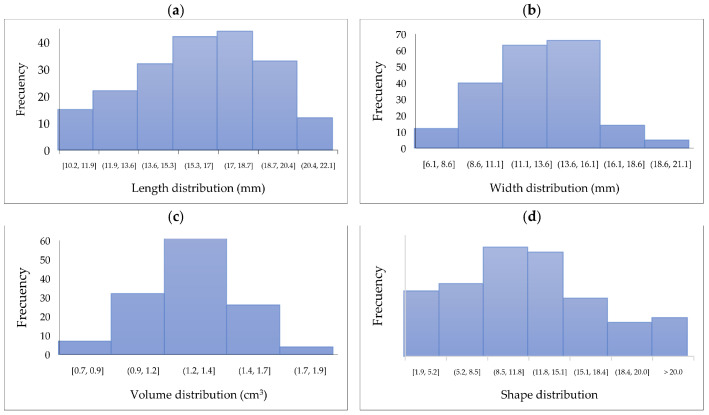
Distribution of berry (**a**) length (mm), (**b**) width (mm), (**c**) volume (cm^3^), and (**d**) shape.

The characterization of length, width, volume, and shape in the 200 berries evaluated is shown in Figure 2. It is observed that most berries have a length between 11.9 and 20.4 mm, while the width is between 8.6 and 16.1 mm, similar to the blueberry width (11–19 mm) [30]. It is essential to note that these berries could change these dimensions with external factors such as environment, soil, and nutrients [31]. The volume distribution shows uniformity, highlighting the values from 8.5 to 15.1 mm^3^, while the shape ranges between 0.9 and 1.7 mostly. As a comparison, the mature blueberry fruits have a shape of 0.91 [32].

**Figure 3:**
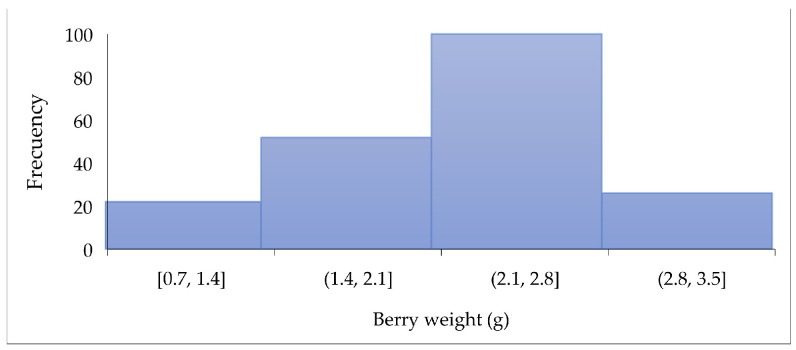
Distribution of berry weight (g).

Figure 3 shows the distribution of weight in the 200 SPF berries evaluated. Most berries weigh between 1.4 and 2.8 g, like the blueberry weight (0.5–3.5 g) [30].

The soluble solids measured as Brix degrees and pH of berries are reported in Table 1. The analysis of SPF berries highlights the importance of ◦Brix because it indicates sweetness and overall fruit quality. The obtained data reveal considerable variation in soluble solids content across samples and reflect differences in ripening stages and environmental influences [10]. The functional implications of °Brix levels extend beyond taste, as sugar content can influence energy density, processing requirements, and the nutritional profile of fruit-derived products. High °Brix fruits require less added sugar in processed foods, enhancing their natural appeal and reducing production costs. Moreover, understanding the relationship between °Brix levels and bioactive compounds such as phenolics and antioxidants could add value as a functional food ingredient. These findings underscore the fruit’s dual potential for consumer markets and industrial applications, affirming its role as a versatile and valuable crop in the food and nutraceutical sectors [10]. The Brix levels found in SPF berries suggest an identifiable pattern of rising sugar content with ripening [15]. The Brix results analyzed by a bilateral samples *t*-test indicated that OM (m = 8.43, sd = 0.18) scored significantly higher than the CM (m = 7.90, sd = 0.18) with a *p*-value of 0.0226.

Likewise, in pH results, it was observed that the OM group scored significantly more than the CM group (*p*-value = 0.001). These changes lead to an increased sugar concentration, which adds to the sweetness of the fruit by bioconversion [15]. Blueberry berries typically have a Brix of 8.5–10.8°, indicating that SPF berries are slightly less sweet. On the other hand, the pH value suggests that it is an acidic fruit like a grape (3.2) and a lemon (3.1) [33].

#### 3.1.2. Color

**Figure 4:**
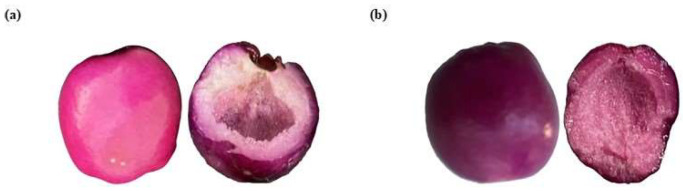
Berries in (**a**) ripening stage 4 with 100% magenta epicarp or consumer maturity (CM) and (**b**) ripening stage 5 with purple epicarp or over-maturity (OM).

Regarding the optical properties, skin berries have more saturated colors than the pulp, while the CM pulp has less intense colors, sometimes close to the white area (Table 2). CM berries are in the magenta zone (350°) in the CIE*L*a*b* space, while OM berries are in the purple zone (Figure 4). Furthermore, in young green fruits (CM), the hue is high (~355° magenta–purple); with ripening, it decreases to values close to 3–50° (red–orange). The intensity of the color increases with maturation due to the degradation of chlorophylls and the increase in pigments such as anthocyanins or carotenoids; however, over-ripeness can be reduced by anthocyanin degradation or browning. In general, the ripening of berries in the Myrtaceae family (such as *Eugenia* spp., *Myrciaria* spp., and *Syzygium* spp., among others) involves changes in the color parameters L* (luminosity), h° (hue), and C* (chroma or saturation). These changes are related to the accumulation of pigments such as chlorophylls, carotenoids, and anthocyanins. The values obtained in this study were compared with studies in wild Andean blueberry [33], Rowan berry [34], Arazá [35], or Champa [34].

The capital letters (A and B) represent the stage of ripeness, while the lowercase letters (a and b) correspond to peel and pulp. When the same capital letter appears, there is no statistically significant difference between ripeness stages; however, the presence of different capital letters denotes an important difference. Similarly, for the lowercase letters, no difference exists when they are the same, but significant differences are observed when they differ.

During the ripening of SPF berries from CM to OM, the peel does not vary significantly in L*. Still, the hue (h*) shifts towards a warmer red, concerning the pulp, which becomes lighter (L* increases), loses red intensity (C* decreases), and gains yellow tones, which represents a clear modification in color, especially in the interior of the fruit. In general, these changes result from biochemical processes (for example, the formation of anthocyanins to the detriment of flavonoids and the appearance of carotenoids or non-colored flavonoids) linked to the sensory and functional quality of the ripe fruit.

### 3.2. Pulverized SPF Berries Characterization

#### 3.2.1. Proximate Composition

The proximal analysis of the SPF berries is shown in Table 3. This fruit has high moisture and low carbohydrate content. In addition, it does not contain fat, and its consumption provides a significant amount of dietary fiber.

#### 3.2.2. Scanning Electron Microscopy (SEM) Analysis

**Figure 5:**
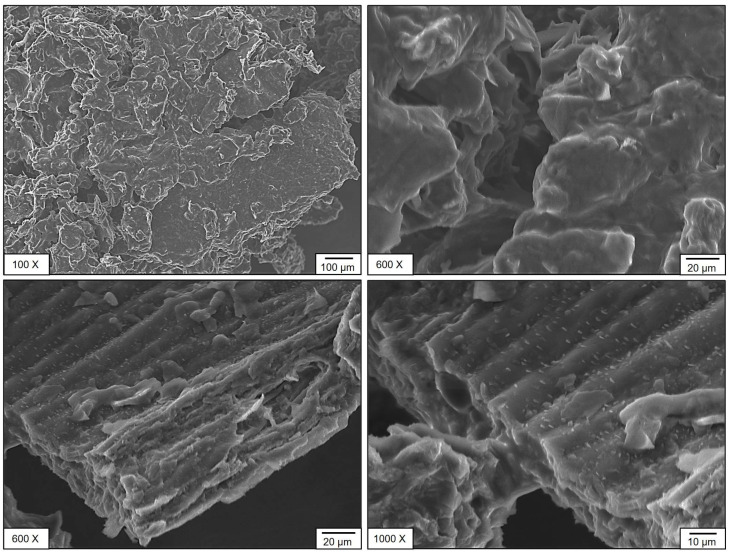
Sem CM stage of maturity of SPF berries.

**Figure 6:**
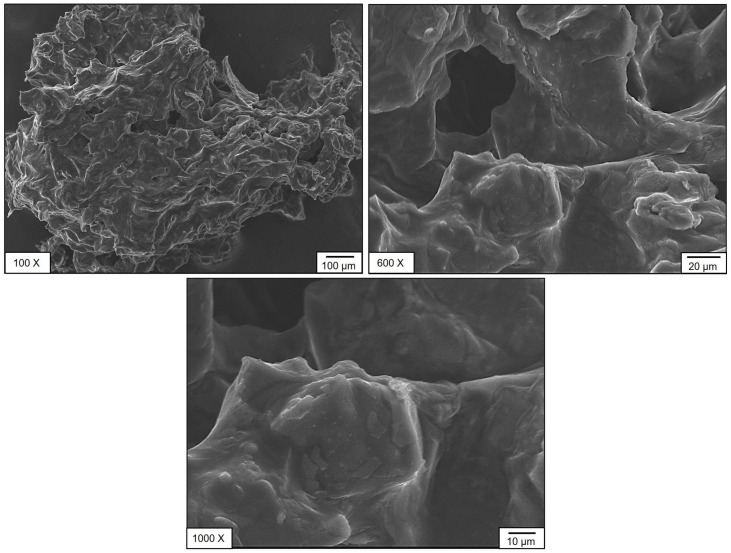
Sem OM stage of maturity of SPF berries.

The microstructural features of SPF berries (bush cherry) samples of CM and OM were analyzed using scanning electron microscopy (SEM) (Figure 5 and Figure 6). Distinct morphological differences were observed between the two compositions. The sample CM exhibited an irregular surface morphology with pores and, in certain regions, a tube-like structure accompanied by granular crystal deposits on the surface (Figure 5). In contrast, sample OM displayed a more uniform structure with a larger average pore size (Figure 6). Notably, both samples exhibited an interconnected pore network, which may contribute to their nutritional functional properties. This influence is mainly observed in phenolic compound degradation. When evaluating the antioxidant capacity of SPF berries, according to the DPPH radical reducing process, the noted matrix degraded during diluted stages, but these might be conserved by bioactive compounds naturally present on the samples or even by different compounds that might be formed from the degradation process during storage time [35].

SEM analysis was performed on dried fruit powders (CM and OM). Evidently, tissues with their original cellular structures were not observed; particle morphology and the most notable surface characteristics were evaluated. In CM, particles with more defined edges and somewhat rough surfaces were identified, suggesting a tissue with greater structural strength. In contrast, in OM, the particles presented more irregular shapes, smoother surfaces, and some evidence of collapse, perhaps caused by the solubilization of sugars and pectin during over-ripeness. This, however, is an indirect approximation of the relationship between ripeness and structural integrity after drying. This could have implications for the technological properties of the powdered product, such as flowability, hygroscopicity, or rehydration, an important consideration when developing new products.

The presence of pectin is related to peaks around 1700 cm^−1^, related to OH^−^: 1740–1700 cm^−1^ associated with C=O of carboxylates: 1200–950 cm^−1^ vibrations of C-O-C and flexion groups around 1400–1300 cm^−1^. Also, the presence of picks around 2800–2950 cm^−1^ related to stretching vibration of CH_2_–CH_3_ groups: 1300–1400 cm^−1^ associated with CH_2_ flexion: 1100–1200 cm^−1^ related to C-O-C vibration and C=O around 1600 cm^−1^ suggested the decrease in pectins of CM to OM stages, and this the reason of texture change of SEM analysis. During ripening, all tissues undergo modifications related to the cell wall, including increased porosity and loss of homogeneity in the structure.

#### 3.2.3. Phytochemical Analysis

##### High-Performance Liquid Chromatography (HPLC)

The results of the High-Performance Liquid Chromatography (HPLC) analysis are shown in Table 4. The extracts with ethanol (SPF EtOH) and water (SPF H_2_O) differ in composition and the ratios of the different components. The significant peaks corresponded to the phenolic acids, flavonoids, and condensed tannins, which have marked antioxidant activities; these results concur with the study of Li et al. [36] in blueberry and strawberry fruits. The ethanol extract (SPF EtOH) showed five primary peaks at 4.940 min, 5.093 min, 6.383 min, 19.040 min, and 23.130 min due to gallic acid, chlorogenic, hydroxycinnamic, ferulic acids, quercetin, rutine, and condensed tannins, respectively. In a study of grape pomace extracts, the authors reported several phenolic compounds, including gallic acid, chlorogenic acid, and ferulic acid [37]. These high antioxidant and antimicrobial activities indicate that ethanol is a suitable solvent for extracting phenolic compounds of low and high polarity. Adding quercetin and rutin at 19.040 min indicates flavonoid fusion and is responsible for its anti-inflammatory and cardioprotective effects. The 14.84% relative area for condensed tannins at 23.130 min mostly explains the extract’s ability to trap free radicals.

On the other hand, the water extract (SPF H_2_O) possessed three significant peaks at 4.937 min, 5.070 min, and 25.150 min, which corresponded mainly to gallic acid (32.41%), caffeic acid (25.43%), and anthocyanin (9.74%). Using the aqueous extraction method allows for the extraction of hydrophilic phenolic acids, where gallic acid is the most prevalent. The presence of bioactive hydroxycinnamic acids, known to possess neuroprotective and anti-inflammatory properties, is reconfirmed by the high percentage of caffeic acid. The anthocyanin peak at 25.150 min also indicates that the water extraction method effectively isolates water-soluble pigmented flavonoids, which are essential for the antioxidant activity of the fruit.

From the analysis of both extracts, it was clear that ethanol is better at extracting flavonoids and tannins, while water favors simpler phenolic acids and anthocyanin extraction. Hence, it can be assumed that SPF EtOH would be more useful in situations where potent antioxidant and antimicrobial activities are needed. At the same time, SPF H_2_O could be used in mixtures where anti-inflammatory and neuroprotective effects are targeted. The different extracts obtained, containing highly bioactive phenolics, confirm the usefulness of SPF berries as a source of natural antioxidants. These observations confirm the pharmacological importance of the fruit and its nutraceutical potential, thus calling for more investigation concerning its bioavailability and interactions in complex biological systems.

The profiles attributed to CM and OM stages show pronounced differences in the composition of bioactive compounds as the fruit matures. The results obtained from High-Performance Liquid Chromatography (HPLC) suggest that polyphenols and their flavonoid derivatives change quite profoundly with the ripening of the fruit [38]. On the other hand, the CM stage shows a distinct profile with a peak at approximately 3.75 min, which might correspond with the biosynthesis time of a significant bioactive substance. With time, small peaks are seen from 6.0 to 8.0 min, which seems to correspond with soft clusters of front-stage flavonoids, phenolic acids, and/or tannins during the earliest stage of their biosynthesis. Since the fruit is still early in polyphenol development, the narrow distribution of peaks indicates that simple phenolic compounds are preferentially present. The chromatogram of OM exhibits a more complex profile with a dominant peak at the time of appearance of 3.973 min (60.92% of the relative area), typical of increased accumulation of polyphenols. Furtive peaks are registered at 4.530 min, 5.063 min, 6.823 min, and 6.980 min. This behavior suggests other complex flavonoids, anthocyanin derivatives, or condensed tannins. It is predictable that the more complicated the anthocyanins and flavonoids are, the more these peaks become pronounced, along with the observed changes in color and antioxidant properties in the ripening phase of the fruit.

##### Total Polyphenol Content (TPC)

Total polyphenol content in CM vs. OM SPF berries was carried out by Folin–Ciocalteu assay; therefore, results are shown in Table 5. It is illustrated that SPF OM fruits exhibited 8.3 ± 0.6 mg GAE per g fresh weight, whereas SPF CM fruits had 6.8 ± 0.3 mg GAE per g FW (mean ± SD, *n* = 3). The TPC results analyzed by a bilateral samples *t*-test indicated that the differences between CM and OM means are equal to zero with a *p*-value of 0.1019. This represents roughly a minimal increase in TPC from the slightly less ripe stage to the fully ripe stage.

The observed minimal increase in TPC from CM to OM suggests that as SPF reaches full maturity, it either synthesizes new phenolic compounds or concentrates them due to water loss or compositional changes [39]. In addition, the contribution to the TPC in OM is the accumulation of anthocyanin pigments because OM fruits have a deep purple coloration, indicating abundant anthocyanins. In contrast, CM fruits, though still pigmented, are comparatively lighter in color as they accumulate during the final ripening; they would register in the Folin–Ciocalteu assay and raise the total phenolic reading [38]. This aligns with the trend reported in other fruit studies; for instance, in tomatoes, the total phenolic content was found to be greater in the red ripe stage relative to the unripe stage, so in our case of an anthocyanin-rich fruit, the increase is plausibly even more directly tied to pigment formation [40].

It is identified that not all polyphenols necessarily increase during ripening in fruit, yet some may decrease because some fruits have shown a net decline in TPC as they ripen according to their initial phenolics, such as tannins or catechins; degradation or dilution by fruit growth occurs [41]. SPF berries might follow a mixed scenario; early in ripening, high levels of tannins are present (contributing to astringency), and as the fruit matures, these may partially diminish (reducing astringency), but simultaneously, anthocyanin content surges [39].

Compared to the literature, the magnitude of TPC observed in SPF is in the same order as that of other antioxidant-rich fruits. As expressed on a fresh weight basis, the OM fruit had about 9 mg GAE per g, equivalent to 900 mg GAE per 100 g fresh fruit, which represents a relatively high amount and underlines the fruit’s potency as a source of polyphenols. For instance, in related Syzygium species (*Syzygium cumini*), the edible pulp was reported to have TPC values on the order of tens of mg GAE/g measured in ethanol extracts, so it supports the idea that Lilly Pilly fruits are similarly rich [42].

The increase in TPC at OM likely correlates with enhanced antioxidant activity of the fully ripe fruits, so polyphenols, especially flavonoids and anthocyanins, are potent antioxidants, and their concentration often determines the radical-scavenging ability of fruit extracts with higher antioxidant power than CM. Generally, a higher TPC is understood as a higher reducing capacity and free-radical quenching ability. The literature supports this connection: for instance, in raspberry fruits, stages with higher total phenolics showed stronger DPPH and ABTS radical scavenging, whereas anthocyanin content alone did not fully explain antioxidant activity [40]. From a nutritional point of view, consuming the fruit at full ripeness might provide health benefits due to the elevated polyphenol content, and it agrees with SPF berries’ reputation as a phytochemically dense fruit.

##### Antioxidant Activity

- DPPH (2,2-Diphenyl-1-picrylhydrazyl)

The results of DPPH (Table 5) show an increase in antioxidant activity between ripeness stages. The DPPH results analyzed by a bilateral samples *t*-test indicated that OM scored significantly higher than the CM with a *p*-value of 0.00001. Fruits at SPF OM exhibited an inhibition of 61.9 ± 1.8%, which was significantly higher than the 46.4 ± 1.8% recorded for SPF CM, so this increase is consistent with the visual and biochemical characteristics. At SPF OM, the fruit displays a deeper purple coloration, indicating higher anthocyanin content, contributing significantly to antioxidant potential. During over-ripening, there is greater availability of compounds capable of donating electrons (anthocyanins or free phenols), which could be related to the degradation of the cellular matrix, which, in turn, releases previously bound compounds. Anthocyanins are potent radical scavengers, and their concentration tends to rise during the final phases of ripening. Furthermore, other polyphenols such as catechins, gallic acid, and flavonoids may also increase during ripening, contributing synergistically to the higher inhibition values observed at SPF OM [40,43].

The trend observed aligns with previous findings in related species such as *Syzygium cumini*, where antioxidant activity increased with ripeness due to anthocyanin accumulation. Thus, the enhanced DPPH inhibition in OM fruit reflects not only pigment development but also a broader biochemical enrichment in phenolic compounds with ripening [43].

b. ABTS [2,2′-Azino-bis (3-ethylbenzothiazoline-6-sulfonic acid)]

The percentage inhibition for both CM and OM was evaluated in concordance with the ABTS radical. As shown in Table 5, the ABTS results analyzed by a bilateral samples *t*-test indicated that OM scored significantly higher than the CM with a *p*-value of 0.0167, suggesting a better radical scavenging effect. This is due to the higher amounts of phenolic compounds, flavonoids, and anthocyanins at early maturity. The strong antioxidant response in OM agrees with previous studies on Syzygium species, which have shown that less mature than overmature fruit contain a greater concentration of polyphenols.

The decrease in ABTS free radical scavenging activity seen in CM indicates that the remaining savored fruits are likely shriveled, and phenolic and anthocyanin contents are severely deficient, most probably due to oxidative enzymatic reaction and metabolic modification of the primary metabolites into secondary ones. The trend observed is coherent, so during over-ripening and transformation of the fruit, the antioxidant activity is often reduced.

The elevated ABTS activity noted in OM indicates that phenolic acids and flavonoids owe a considerable amount of their ability to scavenge radicals to the accompanying anthocyanins. Gallic acid and caffeic acid dominate at early stages and are called powerful antioxidants. Their degradation in fruits ripening in a more advanced state appears to cause an increase in gallic acid, leading to less antioxidant effectiveness and a lower ABTS inhibition percentage. The results support the conjecture that SPF individuals are prime sources of abundant bioactive compounds, which can be utilized in nutraceutical and functional foods. These findings are consistent with past research establishing a direct relationship between polyphenol concentration and the antioxidant activity of Syzygium berries.

Similar to DPPH, the ABTS method shows greater antioxidant capacity in OM. ABTS is more versatile and can detect both water and fat-soluble compounds, indicating that there are more accessible and reactive compounds in OM. The results lead us to the idea that the SPF of higher maturity is a source of large quantities of bioactive compounds, which can be used in functional foods.

Phytochemical analysis reveals that the maturation of SPF berries involves biochemical changes that influence their phytochemical profile and antioxidant capacity. In CM, it presented a higher content of total flavonoids (26.5 ± 1.41 mg/g). In comparison, in OM, they decreased (16.7 ± 0.67 mg/g), suggesting enzymatic degradation or transformation of flavonoids into other structures (phenolic type).

Simultaneously, TPC increased in OM (8.3 ± 0.6 mg _GAE_/g) compared to CM (6.8 ± 0.3 mg _GAE_/g), probably due to the accumulation of other phenolic compounds (non-flavonoids), especially anthocyanins, which specifically increased in OM (571 ± 0.41 mg/100 g _DW_) compared to CM (346 ± 0.35 mg/100 g _DW_), and which, in turn, was reflected in an increase in the intensity of coloration (ripe berries).

This compositional transformation was reflected in the antioxidant capacity. DPPH, FRAP, and ABTS assays revealed increased antioxidant activity in OM. The % DPPH inhibition increased from 46.4 ± 1.8% for CM to 61.9 ± 1.8% for OM, as did the ABTS inhibition (67.89 ± 1.78% for CM to 82.45 ± 1.52% for OM). Finally, the FRAP reducing power increased from 511.2 ± 1.34% to 803.4 ± 1.41% for CM and OM, respectively. During the ripening of SPF berries, the accumulation of phenolic compounds with high antioxidant capacity, mainly anthocyanins, is favored, reinforcing their functional potential for developing healthier foods. However, the reduced flavonoids in overripening suggest that any product development should align with the final product’s nutraceutical or technological objective.

c. FRAP (Ferric Reducing Ability of Plasma)

The FRAP assay determined Eugenia berries’ antioxidant capacity at different maturity levels. The findings are detailed in Table 5. It can be noted that the FRAP results analyzed by a bilateral samples *t*-test indicated that OM scored significantly higher than the CM with a *p*-value of 0.00002. The analysis of CM’s mean FRAP value of 803.40 μmol Trolox/g is far greater than that of CM, 511.20 μmol Trolox/g. There is an apparent reality where it is believed that antioxidant activity is reduced with the allowance of fruit maturation. The trend parallels the previous spectrophotometric assays, showing higher total phenolic contents in ripe fruits, and HPLC profiles that reveal stronger anthocyanin peaks at OM.

As the fruit ripens, the pool of antioxidant phenolics and pigments grows, driving the FRAP increase. Hence, OM extracts also showed stronger DPPH and ABTS radical-scavenging activity than CM, indicating uniformly higher antioxidant capacity in ripe fruits [44]. Therefore, this concordance aligns with the literature: for instance, FRAP, DPPH, and ABTS values track together in phenolic-rich *Eugenia* fruits, all correlating strongly with total phenolics and anthocyanins [45]. These findings underscore that different assays of FRAP vs. radical scavenging give consistent trends when the underlying phenolic content rises.

A higher amount of FRAP at the OM stage can be attributed to increased phenolic complexity and pigment accumulation, accompanied by accumulation of tannins and anthocyanins, each bearing multiple hydroxyl groups capable of reducing Fe^3+^ [46]. As the fruit matures, polymerization and conversion of these compounds into pigmented anthocyanins boost the reducing power [46,47]. Thus, the red pigments and associated flavonoids accumulating by OM are primarily responsible for the enhanced FRAP activity, so the obtained results agree with prior studies of berries and Myrtaceae fruits [47,48]. For instance, the antioxidant activity of raspberries correlates very strongly with total phenolic content and emphasizes that the broad phenolic pool, including complex tannins, underlies FRAP activity.

As in DPPH, the total reducing capacity (FRAP) is higher in OM, suggesting that compounds with reducing power increase with maturation. Therefore, one might suspect that OM preserves stable lowering compounds.

##### Flavonoid Content

CM and OM fruits have differing concentrations of flavonoids, which are evident from the findings (Table 6). The results analyzed by a bilateral samples *t*-test indicated that CM scored significantly higher than OM, with a *p*-value of 0.0147. The data outlined suggests the total flavonoid content (measured in quercetin equivalents) decreased significantly from 26.5 ± 1.41 mg _QE_/g _DW_ at CM to 16.7 ± 0.67 mg_QE_/g_DW_ at OM, many of the compounds quantified by the general flavonoid assay were more abundant in the earlier stage fruit and dropped as the fruit reached full maturity. In addition, this assay detects certain flavonoid classes like flavonols and flavanols. Still, it may not effectively capture anthocyanins because anthocyanins are indeed flavonoids in structure but do not react strongly in the aluminum chloride method [36,49]. Therefore, the decline in flavonoids might be attributed to the loss or transformation of non-anthocyanin flavonoids (such as flavonols and tannin precursors) during ripening, without accounting for the rising anthocyanins that were measured separately [49,50].

For instance, in *Syzygium cumini*, flavonols achieve their highest levels at early maturity and then decline at fruit ripening, coincident with anthocyanin accumulation [49,51]. In the SPF case, CM contained higher amounts of mildly colored flavonoids, but in stage 5, the fruit’s biochemistry shifts toward anthocyanin production. This can be explained by the competition for precursors in the flavonoid biosynthetic pathway and the physiological need to reduce astringency. The fruit might polymerize tannins and certain flavonols to make the ripe fruit more palatable while simultaneously ramping up anthocyanin synthesis. Thus, OM’s lower total flavonoid content is consistent with a ripening strategy where pigmented anthocyanins replace earlier defensive flavonoids [50,51,52].

These results are consistent with previously reported data on Myrtaceae species, which indicates that flavonoids are more copious in immature fruits because of their protective role against oxidative injury, UV radiation, and herbivory [53,54]. As the fruit ripens, there is a metabolic shift towards more significant biosynthesis of other phenolic compounds (e.g., anthocyanins and tannins) that modulate color and taste [55,56].

##### Anthocyanin Content

Anthocyanin content quantified within SPF berries at two ripeness levels is presented in Table 7. The results, analyzed by a bilateral samples *t*-test, indicated that OM scored significantly higher than the CM with a *p*-value of 2.1048 × 10^−8^. Anthocyanin content increased significantly from 346 ± 0.35 mg/100g DW in CM to 571 ± 0.41 mg/100g DW in OM, more than doubling during the final ripening step.

The deepening color of the fruit visually corroborates this result; SPF at the OM stage exhibits a rich purple–black hue compared to the lighter pigmentation at the CM stage. In addition, anthocyanins are the pigments responsible for red, purple, and blue colors in plants, and their biosynthesis is well-known to intensify during the late ripening of many fruits. For instance, anthocyanins in *Syzygium cumini* are first detectable at the onset of color break (green–pink stage) and then surge dramatically as the fruit transitions from dark purple to full black [44,50,57]. Therefore, the data presented show this pattern, with the most significant anthocyanin increase occurring in the final stage of maturation.

Biochemically, the rise in anthocyanin content is attributed to the activation of the flavonoid pathway branch, leading to the production of anthocyanidin as the fruit ripens. Ripening hormones and developmental signals often upregulate anthocyanin biosynthetic genes, producing rapid pigment accumulation in the skin and pulp [58]. For instance, delphinidin-3-gentiobioside and malvidin-3-laminaribioside have been identified as key pigments imparting the deep purple hue [58], so these anthocyanins are highly conjugated polyphenols with multiple hydroxyl groups, which not only give intense color but also confer strong antioxidant properties.

In berries such as blackberries, total anthocyanin content likewise climbs steeply as the fruit changes from unripe to ripe; for example, the anthocyanin concentration in blackberries can increase several-fold between the underripe and overripe stages [59]. In the case of SPF berries, the increase in anthocyanins from CM to OM demonstrates that the accumulation of pigments characterizes late-stage ripening. This explains the color development and is directly linked to the fruit’s antioxidant capacity.

#### 3.2.4. Spectroscopy

##### Fourier-Transform Infrared (FTIR) Spectroscopy

**Figure 7:**
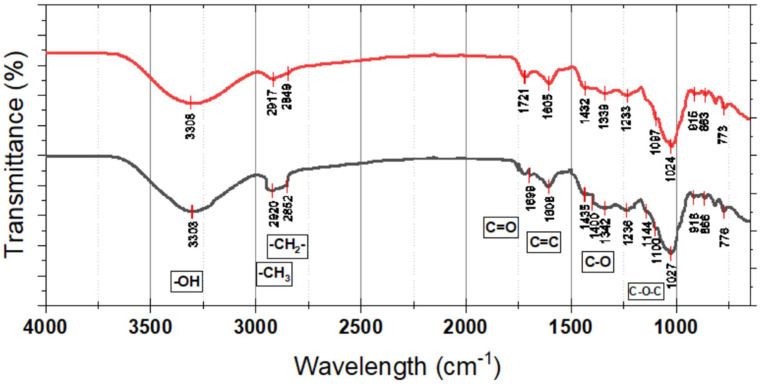
FT-IR results of the sample of *Syzygium paniculatum* Gaertn berries. CM = red; OM = black.

FTIR spectroscopy revealed significant shifts in functional group intensities corresponding to immature and mature fruits (Figure 7). The broad absorption band in the range of 3200–3600 cm^−1^, higher than the region, has been assigned to the stretching vibrations of hydroxyl (O-H) groups characteristic of phenolic compounds. This band was significantly broader and more intense in mature fruits, meaning they contained higher amounts of phenolic compounds, flavonoids, and hydroxylated anthocyanins. Such observation is consistent with chemical assay findings of higher mature phenolic content. Hydroxyl groups are significant for antioxidant activity since they are the source of hydrogen needed for scavenging free radicals. This O-H stretching band is enhanced, indicating that fruit ripening increases not only phenolic concentration but also the ability of the fruit to bear oxidative stress.

Both CM and OM of SPF show remarkable differences in the functional groups’ intensities, which resemble the biochemical changes occurring during ripening. A broad absorption band, which stretches from 3308 cm^−1^ to 3303 cm^−1^, relates to hydroxyl (-OH) group stretching motions, thus suggesting the existence of polymers, flavonoids, or compounds with high amounts of hydroxyl groups. The minor change in position and the dip in intensity at stage 5 indicate some alteration in structure or reduction of hydrogen bonding. Aliphatic C-H stretching vibrations, detected near 2920 cm^−1^ and 2852 cm^−1^ in stage 5, along with 2917 cm^−1^ and 2849 cm^−1^ in stage 4, corresponding to methylene (-CH_2_-) and methyl (-CH_3_) groups, indicate the presence of lipid and carbohydrate components, with minor differences between stages hinting at possible changes in fatty acid composition or cell membrane restructuring. The carbonyl (C=O) stretching at 1721 cm^−1^ in stage 4 and its slight shift to 1699 cm^−1^ in stage 5 suggest modifications in ester or carboxyl functional groups, potentially related to the breakdown of pectin or other esterified compounds during fruit softening.

The maximum peaks at 1605 cm^−1^ and 1608 cm^−1^ are linked to aromatic compounds or polyphenols that bear antioxidant properties for the fruit and are likely to alter with ripening. Hence, the oxidative regions delineate possible changes in their phenolic concentrations. Furthermore, the bands at 1432–1342 cm^−1^ are ascribed to stretching of the C-O bonds and are related to polysaccharides or derivatives of lignin, suggesting that the decline in these concentrations at stage 5 may denote changes in the fruit’s fibrous or carbohydrate structure. Moreover, peaks in the fingerprint region between 1236 cm^−1^ and 773 cm^−1^, such as C-O-C or other ether-type vibrations, signify modifications of carbohydrate chains and glycosidic linkages, which may result from enzymatic cleavage or other processes occurring during ripening.

The spectral features observed through stages CM and OM of ripening suggest the action of myriad simultaneous processes that energetically and chemically mold the fruit from opening to many compositions and possibly functions. The data presented formulates the concept of the phytochemical transformation of SPF as it matures, enhancing the focus on its nutritional and functional benefits.

##### Raman Spectroscopy

**Figure 8:**
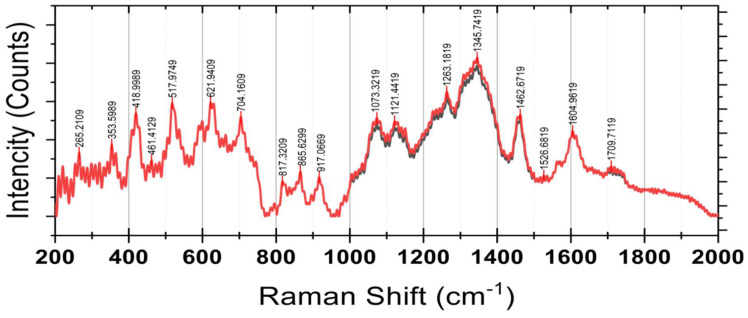

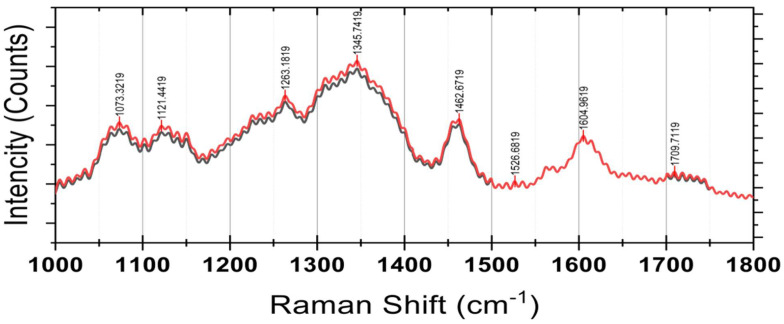
Raman spectroscopy of a powdered sample of *Syzygium paniculatum* Gaertn berries. CM = red; OM = black.

Raman spectroscopy results are shown in Figure 8. The vibration mode at 353 cm^−1^ is primarily related to metal–oxygen bonds or deformation vibrations in aromatic rings. This peak suggests potential interactions between phenolic compounds and residual metal ions in the environment or matrix. Such interactions might form phenolic–metal complexes, contributing to structural stability or antioxidant functionality. This mode also emphasizes the structural role of aromatic rings in phenolic compounds, reinforcing their chemical complexity and potential for interaction with inorganic components. The vibrations at 418 cm^−1^ may relate to either C-C stretching in aliphatic chains or C-O vibrations in aromatic constituents, possibly associated with carbohydrates or glycosides. This mode indicates the presence of ether linkages characteristic of glycosylated phenolics, which designate phenolic compounds conjugated with sugar moieties. Such glycosides facilitate the solubility and bioavailability of water, which are essential functional properties of these compounds in dietary and nutraceutical applications.

The peak verifies the identification of glycosylated phenolics, which refers to carbohydrates associated with phenolic moieties. The 1073 cm^−1^ mode, which is linked to asymmetric C-O-C bond stretching, is mainly associated with ether connections in glycosides, so these conjugates are critical for menaquinone nutraceuticals because they enhance bioactivity and solubility. The vibration at 1121 cm^−1^ is characteristic of C-C bond stretching in the aromatic rings of flavonoids such as quercetin and apigenin. Therefore, the existence of this peak confirms the presence of flavonoids, which have remarkable antioxidant and health benefits for the extract.

The peaks at 1263 cm^−1^ and 1345 cm^−1^ arise from the C-H bending and ring-breathing vibration modes of aromatic substituents like flavonoids and phenolic acids. The increase in intensity at stage 5 indicates a greater concentration of these bioactive compounds, which may contribute to augmenting the fruit’s antioxidant capacity with ripening. Likewise, the peaks 1526 cm^−1^ and 1604 cm^−1^ are due to polyphenol C=C bond stretching in anthocyanins and other pigmented compounds. This behavior reinforces the assumption of a rise in secondary metabolites, which explains the spectral intensification in this region. Signals at lower wavenumbers 265 cm^−1^, 353 cm^−1^, and 418 cm^−1^ are hypothesized to result from skeletal vibrations of polysaccharides and constituents of the cell wall. These bands indicate that structural carbohydrates such as pectins and cellulose are modified during fruit maturation. The peaks in the 700 cm^−1^ to 1100 cm^−1^ region that include but are not limited to 704 cm^−1^, 917 cm^−1^, and 1073 cm^−1^ that are attributed to the C-O and C-C bond stretching of sugars and glycosides suggest that the differences observed between stages results from enzyme induced cleaving or reorganization of carbohydrate frames during fruit ripening.

The spectral region between 1000 cm^−1^ and 1800 cm^−1^ is particularly significant, corresponding to key functional groups associated with polyphenols, carbohydrates, and other biomolecules. The remarkable peaks detected at 1073 and 1121 cm^−1^ will likely be C-O stretching vibrations ascribed to carbohydrates and glycosidic bonds. The intermediate changes between the two stages are possible changes in sugar metabolism or structural polysaccharides during fruit ripening. The C-H bending and the aromatic ring breathing modes at 1263 cm^−1^ show evidence of phenolic compounds. Conversely, the increased intensity during stage 5 indicates a greater concentration of these bioactive compounds, which may possess more potent antioxidant activity.

Shifts in intensity at the 1345 cm^−1^ band, associated with flavonoid and lignin-like structures, imply cell wall adjustments or metabolic remodeling in response to ripening. Approximately 1462 cm^−1^ and 1526 cm^−1^ peaks are attributed to C=C bonds’ stretching vibration in polyphenolic pigments and anthocyanins. These regions show increased intensity, which indicates the build-up of flavonoid pigments and anthocyanins, which may be responsible for the color and antioxidant properties of the fruit. The signal at 1709 cm^−1^ could be from conjugated carbonyl groups due to flavonoids, carbon skeletons, or other derivatives, such as secondary metabolites. These spectral changes convey the changes in phenolic and carbohydrate compounds, suggesting biochemical processes responsible for fruit ripening.

The analysis of the vibrational spectra of SPF berries’ extracts illustrates the intricacy and fragmentation of the chemical constituents. The bioactive properties of the fruit are further confirmed by the presence of primary peaks associated with the phenolic compounds, flavonoids, and their derivatives. The spectral variation between stages 4 and 5 of maturity also demonstrates the remarkable chemical transformation that occurs during the ripening of the fruit. A new potential for antioxidant activity accompanies an increase in polyphenolic signals, while changes in carbohydrate signals suggest physiological changes due to fruit softening and sugar metabolism.

#### 3.2.5. Elemental Analysis by Atomic Absorption Spectroscopy (AAS)

Atomic absorption spectroscopy (AAS) analysis of SPF berries reveals a diverse and nutritionally significant metal profile, offering insight into the potential of the fruit as a dietary source of important minerals [60]. Table 8 illustrates the variations in essential and trace metal concentrations in SPF according to CM and OM ripeness, emphasizing shifts in mineral composition as the fruit progresses through ripening. A powerful pattern observed is the reduction in calcium (Ca), dropping from 120.5 mg/kg in stage 4 to 110.3 mg/kg in stage 5. This decline suggests that calcium may be redistributed or utilized in processes related to cell wall restructuring during maturation [61,62,63]. In the same way, magnesium (Mg) decreases, potentially reflecting its involvement in fundamental physiological adjustments as the fruit ripens [63]. On the other hand, potassium (K) rises from 180.3 mg/kg to 190.7 mg/kg. This trend aligns with its known functions in osmotic regulation and sugar transport processes that generally amplify in mature fruits [64]. In this case, for SPF micronutrients, iron (Fe) and zinc (Zn) exhibit minor increases in OM, probably associated with enzymatic activities that become more predominant in the later stages of ripening, similar to Red Jambo [65].

A slight elevation in sodium (Na) may indicate changes in ion exchange or an improved ability of the fruit tissue to retain water. Meanwhile, manganese (Mn) [66] and copper (Cu) remain relatively stable, suggesting minimal metabolic alterations in their roles [67]. The concentrations of potentially harmful metals, lead (Pb) and cadmium (Cd), stay low, supporting the fruit’s safety for consumption [68,69]. A dilution result may define a slight reduction in Pb and Cd at stage 5 as the fruit gains more biomass. These mineral fluctuations reflect the physiological and biochemical changes attending fruit ripening, so the potassium increase suggests an increase in metabolic activity, which plays a crucial role in fruit softening and sugar accumulation.

In contrast, the decline in calcium may be linked to modifications in cell wall composition. These findings offer noteworthy insights into the nutritional details of SPF at different ripening stages, contributing to better learning of their dietary value [69,70]. However, safety considerations must accompany the nutritional benefits. While the concentrations of barium and other metals remain within safe limits for consumption, continuous monitoring is essential for large-scale applications to prevent potential toxicity. It is crucial when processing the fruit into concentrated forms, as the relative concentration of these elements may increase. Regular quality assessments and adherence to safety standards are necessary to ensure the fruit remains safe for consumption across diverse applications.

In conclusion, the AAS analysis of SPF berries demonstrates a diverse mineral profile that does not support its potential as a good source of minerals. Further research should explore these metals’ bioavailability and synergistic effects on human health, particularly in the context of functional foods and nutraceuticals [57]. The data also emphasize the importance of safety monitoring, ensuring that the fruit’s benefits can be harnessed effectively and responsibly in dietary applications. Finally, the calcium, magnesium, and potassium content can influence the texture and fruit quality. Therefore, treatments with calcium salt could be applied.

## 4. Conclusions

These investigations give an in-depth assessment of the SPF berries’ chemical composition, structural characteristics, and antioxidant potential at two different maturity stages. As observed, ripening is a main phytochemical profile component, which was verified by higher phenolic compounds, increased °Brix, and better antioxidant activity. Ripeness is associated with the elements present in fruits; it is a key variable when determining their nutritional and functional value, which is critical for their use in the food and nutraceutical sectors. The Soxhlet extraction technique isolated bioactive compounds and confirmed its efficiency in retrieving phenolics and flavonoids.

However, alternative methods could further optimize the yield. Hence, the correlation between antioxidant activity and phenolic concentration highlights the potential of SPF as a natural source of antioxidants, capable of contributing to oxidative stress reduction. Structural analyses confirmed biochemical transformations during ripening, particularly in hydroxyl and carbonyl functional groups, supporting the idea that the maturity stage affects molecular composition.

In this study, it was notable that SPF berries have proven to be a valuable functional food candidate with possible uses in health products, drug formulations, and food preservation. However, there is still a lack of other research on its bioavailability to find a significant level of biomolecule integrity in processing and storage over extended periods.

## Figures and Tables

**Table 1:** Mean values ± standard deviations of berry soluble solids and pH at different ripeness stages.

Sample	°Brix	pH
CM	7.90 ± 0.18	3.21 ± 0.01
OM	8.43 ± 0.18 *	3.28 ± 0.01 *

CM: stage 4 of maturity; OM: stage 5 of maturity. * Values are significantly different from the sample by the *t*-test (*p* < 0.05).

**Table 2:** Mean values ± standard deviations of berries’ optical properties (L*, a*, b*, C*, and h*).

Sample	Ripening State	L*	a*	b*	C*	h*
Peel	CM	32.60 ± 2.74 ^aA^	30.50 ± 3.88 ^aA^	−1.97± 1.38 ^aB^	29.55 ± 3.08 ^aA^	356.92 ± 2.43 ^aA^
	OM	32.96 ± 3.15 ^bA^	27.46 ± 2.62 ^aA^	2.04 ± 0.93 ^bA^	27.77 ± 2.60 ^aA^	3.48 ± 0.93 ^bB^
Pulp	CM	39.70 ± 4.53 ^aB^	22.13 ± 3.17 ^bA^	−1.71 ± 0.89 ^aB^	21.70 ± 3.12 ^bA^	355.65 ± 2.39 ^aA^
	OM	50.32 ± 6.05 ^aA^	9.70 ± 3.07 ^bB^	6.22 ± 2.46 ^aA^	10.83 ± 2.87 ^bB^	42.78 ± 8.60 ^aB^

CM: stage 4 of maturity; OM: stage 5 of maturity. Different lowercase letters represent significant differences by ripening state (CM or OM), and capital letters represent significant differences by sample (peel or pulp) (*p* < 0.05).

**Table 3:** Mean values ± standard deviations of berries’ nutritional characterization.

Parameter	Units	Value
Moisture	%	88.9 ± 0.2
Total solids	%	11.1 ± 0.1
Total dietary fiber	%	3.56 ± 0.02
Total protein	% (N × 6.25)	0.627 ± 0.001
Fat	%	0
Ash	%	0.279 ± 0.001
Total carbohydrates	%	7 ± 0.1

**Table 4:** Analysis of SPF extracts with different extraction methods.

Extraction Method	Retention Time (min)	Relative Area (%)	Associated Compounds
In EtOH	4.940	13.82	Phenolic Acids (Gallic, Chlorogenic)
	5.093	12.57	Hydroxycinnamic Acid
	6.383	11.13	Ferulic Acid
	19.040	13.77	Flavonoid (Quercetin, Rutin)
	23.130	14.84	Condensed Tannins
In H_2_O	4.937	32.41	Phenolic
	5.070	25.43	Caffeic
	25.150	9.74	Anthocyanins
CM Stage	3.75	70.12	Gallic Acid/Phenolic Compound
	6.10	12.45	Hydroxycinnamic Acid
	7.25	8.34	Ferulic Acid
	8.10	9.09	Simple Flavonoids
OM Stage	3.973	60.92	Gallic Acid/Simple Phenolics
	4.530	10.18	Caffeic Acid
	5.063	6.72	Condensed Tannins
	6.823	19.30	Flavonoids (Quercetin, Rutin)
	6.980	2.88	Anthocyanins

CM: stage 4 of maturity; OM: stage 5 of maturity.

**Table 5:** Mean values ± standard deviations of berries’ antioxidant capacity at different ripeness stages.

Ripeness Stage	Total Polyphenol Content (mg _GAE_/g _FW_)	DPPH (Inhibition%)	ABTS (Inhibition%)	FRAP (µmol_TECA_/g)
CM	6.8 ± 0.3	46.4 ± 1.8	67.89 ± 1.78	511.20 ±1.34
OM	8.3 ± 0.6	61.9 ± 1.7 *	82.45 ± 1.52 *	803.40 ±1.41 *

CM: stage 4 of maturity; OM: stage 5 of maturity. * Values are significantly different from the ripeness stage by the *t*-test (*p* < 0.05).

**Table 6:** Mean values ± standard deviations of berries’ flavonoid content at different ripeness stages.

Ripeness Stage	Flavonoid Content (mg _QE_/g _FW_)
CM	26.5 ± 1.41 *
OM	16.7 ± 0.67

CM: stage 4 of maturity; OM: stage 5 of maturity. * Values are significantly different from the ripeness stage by the *t*-test (*p* < 0.05).

**Table 7:** Mean values ± standard deviations of berries’ anthocyanin content at different ripeness stages.

Ripeness Stage	Wavelength (nm)	Absorbance (A.U.)	Total Anthocyanins (mg/100 g _DW_)
CM	535	0.186	346 ± 0.35346
OM	535	0.307	571 ± 0.41 *

CM: stage 4 of maturity; OM: stage 5 of maturity. * Values are significantly different from the ripeness stage by the *t*-test (*p* < 0.05).

**Table 8:** Mean values ± standard deviations of the berries’ metal concentration at different ripeness stages.

Metal Element	CM (mg/kg)	OM (mg/kg)
Calcium (Ca)	120.5 ± 3.2	110.3 ± 2.9
Sodium (Na)	136.5 ± 0.2	138.3 ± 0.9
Potassium (K)	180.3 ± 4.1	190.7 ± 4.3
Magnesium (Mg)	30.8 ± 1.7	28.4 ± 1.6
Iron (Fe)	4.6 ± 0.3	5.0 ± 0.4
Zinc (Zn)	2.1 ± 0.2	2.3 ± 0.2
Manganese (Mn)	1.5 ± 0.1	1.6 ± 0.1
Copper (Cu)	0.9 ± 0.1	1.0 ± 0.1
Lead (Pb)	0.05 ± 0.01	0.04 ± 0.01
Cadmium (Cd)	0.02 ± 0.005	0.018 ± 0.004

CM: stage 4 of maturity; OM: stage 5 of maturity.

## Data Availability

The original contributions presented in the study are included in the article, further inquiries can be directed to the corresponding authors.

## References

[1] Jiménez-Munoz L.M., Tavares G.M., Corredig M. (2021). Design future foods using plant protein blends for best nutritional and technological functionality. Trends Food Sci. Technol..

[2] Salazar D., Arancibia M., Ocaña I., Rodríguez-Maecker R., Bedón M., López-Caballero M.E., Montero M.P. (2021). Characterization and technological potential of underutilized ancestral andean crop flours from Ecuador. Agronomy.

[3] Galanakis C.M. (2021). Functionality of food components and emerging technologies. Foods.

[4] Acurio L., Salazar D., Guanoquiza I., García-Segovia P., Martínez-Monzó J., Igual M. (2025). Ecuadorian roots flours: Bioactive compounds and processing properties. J. Agric. Food Res..

[5] Chagas M.S.S., Behrens M.D., Moragas-Tellis C.J., Penedo G.X.M., Silva A.R., Gonçalves-de-Albuquerque C.F. (2022). Flavonols and Flavones as Potential anti-Inflammatory, Antioxidant, and Antibacterial Compounds. Oxid. Med. Cell. Longev..

[6] Ruiz Rodríguez L.G., Zamora Gasga V.M., Pescuma M., Van Nieuwenhove C., Mozzi F., Sánchez Burgos J.A. (2021). Fruits and fruit by-products as sources of bioactive compounds. Benefits and trends of lactic acid fermentation in the development of novel fruit-based functional beverages. Food Res. Int..

[7] El Sheikha A.F. (2022). Nutritional Profile and Health Benefits of Ganoderma lucidum “Lingzhi, Reishi, or Mannentake” as Functional Foods: Current Scenario and Future Perspectives. Foods.

[8] Nemzer B.V., Al-Taher F., Yashin A., Revelsky I., Yashin Y. (2022). Cranberry: Chemical composition, antioxidant activity and impact on human health: Overview. Molecules.

[9] Nabeshima E.H., Tavares P.E.R., Lemos A.L.S.C., Moura S.C.S.R. (2024). Emerging ingredients for clean label products and food safety. Braz. J. Food Technol..

[10] Pérez Mora W.H., Mojica Gómez J. (2023). Physicochemical analysis of fruits of Syzygium paniculatum in diff erent stages of maturity. Entre Cienc. E Ing..

[11] de Amorim M.S., Verdan M.H., Oliveira C.S., Santos A.D.C. (2024). Essential Oils of Neotropical Myrtaceae Species From 2011 Until 2023: An Update. Chem. Biodivers..

[12] Cumpston Z. (2019). Indigenous Plant Use: A booklet on the Medicinal, Nutritional and Technological Use of Indigenous Plants.

[13] Saber F.R., Munekata P.E., Rizwan K., El-Nashar H.A., Fahmy N.M., Aly S.H., El-Shazly M., Bouyahya A., Lorenzo J.M. (2024). Family Myrtaceae: The treasure hidden in the complex/diverse composition. Crit. Rev. Food Sci. Nutr..

[14] Maheshwari P., Nair A., Shanmugasundaram P. (2022). An overview on diabetic profile of various Syzygium species. J. Pharm. Negat. Results.

[15] Pachulicz R.J., Yu L., Jovcevski B., Bulone V., Pukala T.L. (2022). Polyphenol characterisation and diverse bioactivities of native Australian lilly pilly (Syzygium paniculatum) extract. Food Funct..

[16] Julizan N., Ishmayana S., Zainuddin A., Van Hung P., Kurnia D. (2023). Potential of Syzygnium polyanthum as Natural Food Preservative: A Review. Foods.

[17] Armijos C., Ramírez J., Vidari G. (2022). Poorly Investigated Ecuadorian Medicinal Plants. Plants.

[18] Ranghoo-Sanmukhiya V., Chellan Y., Soulange J., Lambrechts I., Stapelberg J., Crampton B., Lall N. (2018). Biochemical and phylogenetic analysis of Eugenia and Syzygium species from Mauritius. J. Appl. Res. Med. Aromat. Plants.

[19] da Silva J.P.R., Aragão A.C.R., dos Santos Sousa Junior R., Crisóstomo Bezerra Costa C.A., da Silva Moura O.F., Araújo T.D.S., da Silva D.A., da Silva Neto A.R., Silva K.C., Lopes T.O. (2023). Phytochemical analysis, in vitro antioxidant and anticholinesterase activities of Solanum paniculatum L. and an in-silico test with the AChE enzyme. S. Afr. J. Bot..

[20] Konda P.Y., Chennupati V., Dasari S., Sharma N., Muthulingam M., Ramakrishnan R., Sade A., Jagadheeshkumar V., Natesan V., Jaiswal K.K. (2021). Ethno-pharmacological insulin signaling induction of aqueous extract of Syzygium paniculatum fruits in a high-fat diet induced hepatic insulin resistance. J. Ethnopharmacol..

[21] Amir Rawa M.S., Mazlan M.K.N., Ahmad R., Nogawa T., Wahab H.A. (2022). Roles of Syzygium in anti-cholinesterase, anti-diabetic, anti-inflammatory, and antioxidant: From Alzheimer’s perspective. Plants.

[22] Acurio L., Salazar D., García M.E., García-Segovia P., Martínez-Monzó J., Igual M. (2024). Characterization, mathematical modeling of moisture sorption isotherms and bioactive compounds of Andean root flours. Curr. Res. Food Sci..

[23] Association of Analytical Communities (2023). Official Methods of Analysis.

[24] Nurlely N., Perdana Putra A.M., Nurrochmad A., Widyarini S., Fakhrudin N. (2024). Extraction, phytochemicals, bioactivities, and toxicity of Syzygium polyanthum: A comprehensive review. J. Herbmed Pharmacol..

[25] Rocha Martins G., Ferreira Monteiro A., Lopes do Amaral F.R., da Silva A.S.A. (2021). A validated Folin-Ciocalteu method for total phenolics quantification of condensed tannin-rich açaí (Euterpe oleracea Mart.) seeds extract. J. Food Sci. Technol..

[26] Rahman M., Buragohain R., Das R., Islam M.A., Hazarika N.K., Pathak K., Barman P. (2024). Comparative biochemical evaluation of the proximate content, antioxidant properties, and phytochemical constituents of ethnomedicinally important Docynia indica from Northeast India. Vegetos.

[27] Elhassaneen Y., Elbassouny G., Emam O., Hashem S. (2023). Influence of novel freezing and storage technology on nutrient contents, bioactive compounds and antioxidant capacity of black eggplant. J. Agric. Crops..

[28] Shraim A.M., Ahmed T.A., Rahman M.M., Hijji Y.M. (2021). Determination of total flavonoid content by aluminum chloride assay: A critical evaluation. LWT.

[29] Filip M., Mihaela V., Copaciu F., Coman V. (2012). Identification of anthocyanins and anthocyanidins from berry fruits by chromatographic and spectroscopic techniques to establish the juice authenticity from market. JPC J. Planar Chromatogr..

[30] Jorquera-Fontena E., Génard M., Ribera-Fonseca A., Franck N. (2017). A simple allometric model for estimating blueberry fruit weight from diameter measurements. Sci. Hortic..

[31] Montgomery D.R., Biklé A. (2021). Soil Health and Nutrient Density: Beyond Organic vs. Conventional Farming. Front. Sustain. Food Syst..

[32] Wan X., Wu Z., Sun D., Long L., Song Q., Gao C. (2024). Cytological characteristics of blueberry fruit development. BMC Plant Biol..

[33] Tokunbo Bamise C., Obhioneh Oziegbe E. (2013). Laboratory analysis of pH and neutralizable acidity of commercial citrus fruits in Nigeria. Adv. Biol. Res..

[34] Balaguera-López H.E., Fischer G., Herrera-Arévalo A. (2022). Postharvest physicochemical aspects of Campomanesia lineatifolia R. & P. fruit, a Myrtaceae with commercial potential. Rev. Colomb. Cienc. Hortic..

[35] Santos M., Gonçalves É. (2016). Effect of different extracting solvents on antioxidant activity and phenolic compounds of a fruit and vegetable residue flour. Sci. Agropecu..

[36] Li W., Zhang X., Wang S., Gao X., Zhang X. (2024). Research Progress on Extraction and Detection Technologies of Flavonoid Compounds in Foods. Foods.

[37] Recinella L., Chiavaroli A., Veschi S., Cama A., Acquaviva A., Libero M.L., Leone S., Di Simone S.C., Pagano E., Zengin G. (2022). A grape (*Vitis vinifera* L.) pomace water extract modulates inflammatory and immune response in SW-480 cells and isolated mouse colon. Phytother. Res..

[38] Gibson L., Rupasinghe H.P., Forney C.F., Eaton L. (2013). Characterization of Changes in Polyphenols, Antioxidant Capacity and Physico-Chemical Parameters during Lowbush Blueberry Fruit Ripening. Antioxidants.

[39] Kobori R., Yakami S., Kawasaki T., Saito A. (2021). Changes in the polyphenol content of red raspberry fruits during ripening. Horticulturae.

[40] Antala P.A., Chakote A., Varshney N., Suthar K., Singh D., Narwade A., Patel K., Gandhi K., Singh S., Karmakar N. (2025). Phytochemical and metabolic changes associated with ripening of Lycopersicon esculentum. Sci. Rep..

[41] Pham N.M.Q., Chalmers A.C., Vuong Q.V., Bowyer M.C., Scarlett C.J. (2017). Characterising the Physical, Phytochemical and Antioxidant Properties of the Tuckeroo (*Cupaniopsis anacardioides*) Fruit. Technologies.

[42] Makkar H.P.S. (2003). Quantification of Tannins in Tree and Shrub Foliage: A Laboratory Manual.

[43] Khasanah U., Adiningsih O.R., Anggraeni E.D., Uliyah F.U., Ramadhani A.I., Saraswati A.A.D., Ardiani G.K. (2022). Phytochemical screening, total phenolic content and cytotoxic activity of seed, leaves, and pulp from Syzygium cumini against breast cancer cell culture 4T1. Res. J. Pharmacogn..

[44] Li J., Shi C., Shen D., Han T., Wu W., Lyu L., Li W. (2022). Composition and Antioxidant Activity of Anthocyanins and Non-Anthocyanin Flavonoids in Blackberry from Different Growth Stages. Foods.

[45] Silva J.D.R., Arruda H.S., Andrade A.C., Berilli P., Borsoi F.T., Monroy Y.M., Rodrigues M.V.N., Sampaio K.A., Pastore G.M., Marostica Junior M.R. (2024). Eugenia calycina and Eugenia stigmatosa as Promising Sources of Antioxidant Phenolic Compounds. Plants.

[46] Kim S., Semple S.J., Simpson B.S., Deo P. (2020). Antioxidant and Antiglycation Activities of Syzygium paniculatum Gaertn and Inhibition of Digestive Enzymes Relevant to Type 2 Diabetes Mellitus. Plant Foods Hum. Nutr..

[47] Wang L., Li R., Zhang Q., Liu J., Tao T., Zhang T., Wu C., Ren Q., Pu X., Peng W. (2022). Pyracantha fortuneana (Maxim.) Li: A comprehensive review of its phytochemistry, pharmacological properties, and product development. Front. Sustain. Food Syst..

[48] Lee J., Hwang I., Park Y.-S., Lee D.Y. (2023). Occurrence and health risk assessment of antimony, arsenic, barium, cadmium, chromium, nickel, and lead in fresh fruits consumed in South Korea. Appl. Biol. Chem..

[49] Wathon M.H., Susilowati E., Ariani S.R.D. (2023). Anthocyanins from Java Plum Fruits (*Syzygium cumini*) and their Stability in Various pHs. J. Biomim. Biomater. Biomed. Eng..

[50] Belwal T., Pandey A., Bhatt I.D., Rawal R.S., Luo Z. (2019). Trends of polyphenolics and anthocyanins accumulation along ripening stages of wild edible fruits of Indian Himalayan region. Sci. Rep..

[51] Govindarajan N., Ravichandran L., Chelladurai P.K., Pandey A., Murugesan V., Kusuma G. (2023). Pharmacopoeial Compliance of Marketed Formulations containing seeds of *Syzygium cumini* (L.) Skeels. Res. J. Pharm. Technol..

[52] Gouws C.A., Georgouopoulou E., Mellor D.D., Naumovski N. (2019). The Effect of Juicing Methods on the Phytochemical and Antioxidant Characteristics of the Purple Prickly Pear (*Opuntia ficus indica*)—Preliminary Findings on Juice and Pomace. Beverages.

[53] de Paulo Farias D., Neri-Numa I.A., de Araújo F.F., Pastore G.M. (2020). A critical review of some fruit trees from the Myrtaceae family as promising sources for food applications with functional claims. Food Chem..

[54] Barboni T., Cannac M., Massi L., Perez-Ramirez Y., Chiaramonti N. (2010). Variability of Polyphenol Compounds in Myrtus Communis L. (Myrtaceae) Berries from Corsica. Molecules.

[55] Chirinos R., Galarza J., Pallardel I., Pedreschi R., Campos D. (2010). Antioxidant compounds and antioxidant capacity of Peruvian camu camu (Myrciaria dubia (H.B.K.) McVaugh) fruit at different maturity stages. Food Chem..

[56] da Veiga Correia V.T., da Silva P.R., Ribeiro C.M., Ramos A.L., Mazzinghy A.C., Silva V.D., Júnior A.H., Nunes B.V., Vieira A.L., Ribeiro L.V. (2022). An Integrative Review on the Main Flavonoids Found in Some Species of the Myrtaceae Family: Phytochemical Characterization, Health Benefits and Development of Products. Plants.

[57] Gaibor F.M., Rodríguez D., Fundora L., Salas E., Rodríguez J.L., Falco A.S., Casariego A., García M.A. (2016). Evaluación de las características físicas, químicas, toxicológicas, antibacterianas y sensoriales del cerezo negro (*Syzygium cumini* L.): Physical, chemical, toxicological, antibacterial and sensorial assessment of black cherry (*Syzygium cumini* L.). Cienc. Y Tecnol. De Aliment..

[58] Nguyen N. (2024). Towards a Comprehensive Understanding of the Fruit Development and Ripening Process of Wild Bilberry (*Vaccinium myrtillus* L.). Ph.D. Thesis.

[59] Tosun I., Ustun N.S., Tekguler B. (2008). Physical and chemical changes during ripening of blackberry fruits. Sci. Agric..

[60] Medveckienė B., Kulaitienė J., Vaitkevičienė N., Levickienė D., Bunevičienė K. (2022). Effect of Harvesting in Different Ripening Stages on the Content of the Mineral Elements of Rosehip (*Rosa* spp.) Fruit Flesh. Horticulturae.

[61] Jaime-Guerrero M., Álvarez-Herrera J.G., Fischer G. (2024). Effect of calcium on fruit quality: A review. Agron. Colomb..

[62] Hocking B., Tyerman S.D., Burton R.A., Gilliham M. (2016). Fruit Calcium: Transport and physiology. Front. Plant Sci..

[63] Quddus M.A., Siddiky M.A., Hussain M.J., Rahman M.A., Ali M.R., Taher Masud M.A. (2022). Magnesium influences growth, yield, nutrient uptake, and fruit quality of tomato. Int. J. Veg. Sci..

[64] Clausen T. (2003). Na+-K+ pump regulation and skeletal muscle contractility. Physiol. Rev..

[65] Gibbert L., Bampi M., Kerkhoven N.C., Benevide Sereno A., de Queiroz Pereira Pinto C., Rodrigues Ferreira S.M., Tribuzy de Magalhães Cordeiro A.M., Lins de Albuquerque Meireles B.R., Campelo Borges G.d.S., Meira Silveira J.L. (2022). Nutritional potential of Red Jambo fruit: Dietary fibers, minerals, antioxidant potential, and bioaccessibility of phenolic compounds. Res. Soc. Dev..

[66] Martinez-Finley E.J., Chakraborty S., Aschner M. (2013). Manganese in biological systems. Encyclopedia of Metalloproteins.

[67] Tsang T., Davis C.I., Brady D.C. (2021). Copper biology. Curr. Biol..

[68] Sangeetha V., Dutta S., Moses J., Anandharamakrishnan C. (2022). Zinc nutrition and human health: Overview and implications. eFood.

[69] Behera P.R., Chitdeshwari T., Malarvizhi P., Sivakumar U., Vethamoni P.I. (2021). Zinc (Zn) and Iron (Fe) Fertilization for Improving the Antioxidant Enzyme Activity and Biochemical Constituents in Capsicum Hybrids. Int. J. Plant Sci..

[70] Costa F., de Lurdes Baeta M., Saraiva D., Verissimo M.T., Ramos F. (2011). Evolution of mineral contents in tomato fruits during the ripening process after harvest. Food Anal. Methods.

